# Extrinsic stressors modulate resource evaluations: insights from territoriality under artificial noise

**DOI:** 10.1186/s12983-021-00397-x

**Published:** 2021-03-20

**Authors:** Kyriacos Kareklas, Hansjoerg P. Kunc, Gareth Arnott

**Affiliations:** 1 Integrative Behavioural Biology Group, Instituto Gulbenkian de Ciência, Rua da Quinta Grande 6, 2780-156 Oeiras, Portugal; 2 School of Biological Sciences, Queen’s University Belfast, 19 Chlorine Gardens, Belfast, BT9 5DL Northern Ireland, UK

**Keywords:** Resource value, Contest, Extrinsic stressor, Noise, Territoriality, Nest building

## Abstract

**Background:**

Competition is considered to rely on the value attributed to resources by animals, but the influence of extrinsic stressors on this value remains unexplored. Although natural or anthropogenic environmental stress often drives decreased competition, assumptions that this relies on resource devaluation are without formal evidence. According to theory, physiological or perceptual effects may influence contest behaviour directly, but motivational changes due to resource value are expected to manifest as behavioural adjustments only in interaction with attainment costs and resource benefits. Thus, we hypothesise that stressor-induced resource devaluations will impose greater effects when attainment costs are high, but not when resource benefits are higher. Noise may elicit such effects because it impacts the acoustic environment and imposes physiological and behavioural costs to animals. Therefore, we manipulated the acoustic environment using playbacks of artificial noise to test our hypotheses in the territorial male Siamese fighting fish, *Betta splendens*.

**Results:**

Compared to a no-playback control, noise reduced defense motivation only when territory owners faced comparatively bigger opponents that impose greater injury costs, but not when territories also contained bubble nests that offer reproductive benefits. In turn, nest-size decreases were noted only after contests under noise treatment, but temporal nest-size changes relied on cross-contest variation in noise and comparative opponent size. Thus, the combined effects of noise are conditional on added attainment costs and offset by exceeding resource benefits.

**Conclusion:**

Our findings provide support for the hypothesised modulation of resource value under extrinsic stress and suggest implications for competition under increasing anthropogenic activity.

**Supplementary Information:**

The online version contains supplementary material available at 10.1186/s12983-021-00397-x.

## Background

Contest theory predicts that resource competition, a key driver of natural and sexual selection, is influenced by resource value, with contestants expected to modulate their behaviour according to the perceived or attributed value [1]. This value is typically described in terms of quality, comprising a set of intrinsic factors adding costs or benefits to resources [2–4]. For example, territories with greater food abundance and lower predation risk exhibit increased occupancy by opportunistic birds [5] and the motivation of hermit crabs to compete for shells increases when the size of a prospective shell is more adequate than that of their current shell [6]. However, resource attainment relies on inter-contestant differences in competitive ability factors, such as weaponry, morphology, experience and physiological state [7], which together comprise a contestant’s resource holding potential (RHP [8, 9]). As such, a major contest research focus has been the information-gathering and decision-making strategies used by contenders, where animals may assess their own RHP and compare it to the opponent’s when deciding to engage, respond or quit during contests [7, 10]. Yet, the extent to which the motivation to compete with a particular opponent is co-modulated by the assessment of intrinsic factors that influence resource quality is often unclear [1]. Furthermore, the way that this might be further impacted by extrinsic factors has been largely neglected, with only few recent examples (e.g. water-flow and oxygen-concentration effects on sea anemone territorial contests [11, 12]).

External factors influencing resource choice and use often include biotic and abiotic environmental stressors, deriving from either natural processes or anthropogenic activity. For example, bird territorial occupancy and biodiversity is affected by wind speed, solar elevation and temperature, as well as human presence [13, 14]. Furthermore, fish territory use and distribution is influenced by temperature, acidity and dissolved oxygen fluctuations due to spatiotemporal variation and human activity, but also by predation and competition implicating native and human-introduced invasive species [15, 16]. These and other evidence identifies that both anthropogenic and naturally occurring stressors can drive animals to abandon resources that are otherwise of great fitness benefit, such as moose trading off food-abundant territories for ones with lower predation [17] or elasmobranchs abandoning food-rich areas influenced by natural or human derived changes in temperature or salinity [18]. However, animals have not evolved with the rapid onset of human-derived stressors and their responses can be maladaptive or insufficient to overcome these stressors [19, 20]. Therefore, the ability of animals to reevaluate resources under anthropogenic stressors can be additionally elucidating when examining the coping strategies animals employ under such novel pressures.

**Fig. 1:**
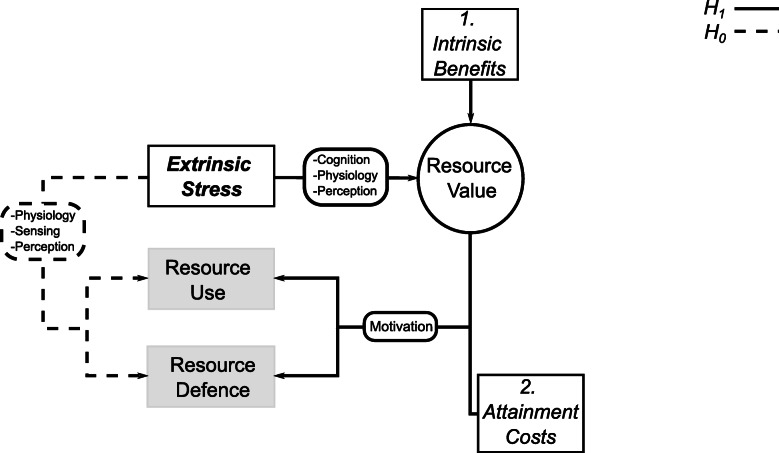
Predicted effects of extrinsic stressors on resource value. The diagram illustrates how changes to resource value are expected to affect resource use and defence by influencing motivation in interaction with other benefits and costs (*H*_*1*_), as opposed to alternatively mediated effects that do not implicate such interactions (*H*_*0*_)

The aforementioned effects of extrinsic stressors on resource attainment and use, added to the identified physiological and behavioral costs imposed by extrinsic stressors (reviewed by Killen et al. [21]), often justify the equivalence of stressor presence with lower resource value, but this is based on the assumption that animals assess extrinsic stressors and modulate their perceived or attributed resource value accordingly (see review by Arnott and Elwood [1]). Yet, there is little evidence that the reduced contest motivation and performance under extrinsic stress identified by some studies [11, 12] is a result of changes in attributed or perceived resource value [1]. Indeed, effects may not rely on cognitive evaluations [22, 23], but instead on direct effects from physiological changes [21] or the disruption and masking of relevant signals from an opponent or the environment [24, 25]. To clarify this, the implication of extrinsic stressors on resource evaluation can be identified by examining interaction effects with attainment costs (e.g. opponent RHP) and added resource benefits (e.g. territory with shelters as opposed to without). According to theory, the motivation to defend and use resources increases with their value and decreases with attainment costs [1, 7, 26]. Thus, the change in resource evaluation due to the presence of stressors is predicted to correspond to changes in motivation, in interaction with contest costs and resource benefits (Fig. 1; *H*_*1*_). In contrast, direct effects from physiology and signal sensing or perception are expected to manifest changes in resource defence and use, independently from such interactions (Fig. 1; *H*_*0*_).

For male Siamese fighting fish, *Betta splendens,* territories are a key resource because they provide space to build bubble-nests for their offspring, which plays a significant role in reproductive success [27–30]. The high fitness value of territory for males looking to build nests, and the even higher value for males protecting existing nests, underlies much of the *B. splendens* well-defined agonistic repertoire, including their display, attack and use of visual cues from opponents to assess their ability (RHP) and compare it to their own [31–36]. Importantly, motivational variation in the use of territory for nest building can be quantified by nest-size changes, which indicate the expansion or maintenance of buoyancy and holding capacity [33]. Also, contest motivation can be accurately quantified via the startle-probe approach, which indicates motivation independently of ongoing behaviour and outcome by quantifying recovery from a startling stimulus. This can be applied at various stages of the contest [37], and has been validated in fish [38, 39] and specifically male *B. splendens* [36]. Consequently, the effects of external stressors on resource value can be identified in this species by measuring motivational changes in territorial defence and bubble-nest construction.

These effects on territory use and defense motivation may be elicited by anthropogenic activities that introduce physiologically and behaviourally costly extrinsic stressors, especially when these stressors are unpredictable (i.e. unfamiliar or novel stressors leading to uncertainty), frequent in occurrence (i.e. having a chronic impact) and widespread [19, 25, 40, 41]. One extrinsic stressor with such characteristics is noise, which influences fish behaviour, physiology and reproduction, by changes to underwater acoustic environments [40]. For instance, noise can impact fish swim-bladder and ear function, cardiac output, and metabolism, by changing pressure and particle motion; it can trigger stress responses by introducing novel sounds and may also limit the perception of predator, prey, conspecific and mate acoustic signals by masking or distraction (see reviews by: Brumm and Slabbekoorn [42]; Slabbekoorn et al. [25]; Kight and Swaddle [40]; Francis and Barber [43]; Kunc et al. [44]). Therefore, noise conditions may be associated with costs that influence the perceived or attributed value of a resource, such as territory. Alternatively, noise may affect the motivation to collect information on resource quality, where in the presence of noise animals spend less time inspecting new better-quality resources and are less likely to swap their current inferior resources for them (e.g. shell acquisition in hermit crabs [45, 46]). Collectively, these effects suggest that changes to underwater acoustic environments may contribute to variation in perceived or attributed resource value, which can influence territorial defence and use.

**Fig. 2:**
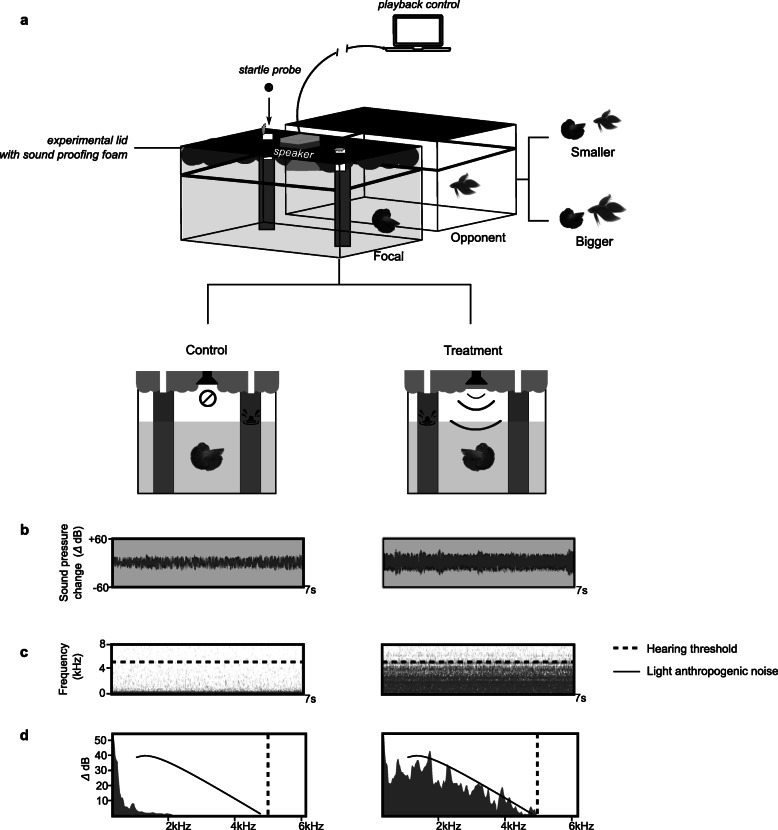
Experimental manipulations during tests. **a** Focals were exposed to staged intrusions by either a bigger or smaller opponent, under a white-noise treatment or a no-noise control (via speaker in experimental lid, in place in all contests); nest presence and size were recorded before contests and aggressive motivation probed via the startle recovery approach using interruptive marble drops through access tubes in the lid. **b** Recordings of underwater sound conditions with a hydrophone indicated greater changes in sound pressure level and **c** increases in wavelength frequency during noise treatment, compared to the control condition. Frequencies ranged up to expected hearing maxima for *B. spelndens* (dashed line: auditory structures and sensitivity; Ladich and Yan, 1998 [47]; Ladich and Popper, 2001 [48]). **d** Underwater sound profiles under noise exhibited sound pressure changes at higher frequencies, which reflected anthropogenic noise near light terrestrial and shipping traffic (full line: adapted from Dahl et al., 2007 [49])

Here we aim to quantify the role of noise in modulating perceived resource value, which is expected to vary with added costs and benefits (Fig. 1: *H*_*1*_). As such, we tested three experimental hypotheses by manipulating the acoustic environment during contests between male *B. splendens*, using playbacks containing artificial white noise (Fig. 2). First, we tested whether artificial noise reduces the motivation of male *B. splendens* to engage in territorial defence, predicting this effect will only be evident when facing relatively bigger opponents, because territories with lower value are less worth protecting when likely injury costs are high. Second, we tested whether fish will be more motivated to defend territories with nests, because nests increase the subjective value of the territory, either due to the energy invested in their construction or due to their positive influence on reproductive success. Following from this, increased background noise was predicted to have a more prominent effect on the motivation to defend territory in the absence of nests, because the costs from increased noise are countered by the benefits to reproductive success by nest presence. Third, we hypothesise that following their exposure to increased noise during contests, fish would be less motivated to use the territory for building nests, due to the fitness costs attributed to noise. Thus, we quantify changes to resource value by examining the interaction between acoustic condition, opponent assessment and nest-building.

## Results

**Fig. 3:**
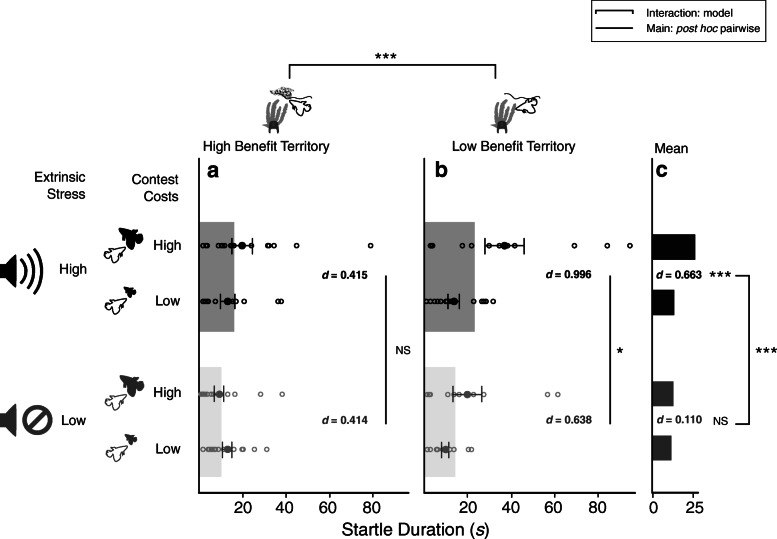
Noise effects on startle duration during contests and interactions with nest presence and opponent size. **a** Startle durations were not affected by acoustic conditions or opponent size when territory included bubble-nests. Conversely, **b** startle duration was shorter under noise when no nest was present, resulting in an effect from the interaction between acoustic condition and nest presence. **c** Interaction effects were also identified between acoustic condition and opponent size, where decreases in motivation (revealed by a longer startle duration) are only noted when facing bigger opponents under noise. Interval bars indicate standard error and effect sizes are indicated by Cohen’s *d* where appropriate. [*P ≤ 0.05, ***P ≤ 0.001]

Startle durations were significantly affected by acoustic condition in interaction with bubble-nest presence (*χ*^*2*^_1, 119_ = 10.08, *P* = 0.001, *d* = 0.546) and relative opponent size (*χ*^*2*^_1, 119_ = 18.09, *P* < 0.001, *d* = 0.890), but not at the three-way interaction (*χ*^*2*^_1, 119_ = 0.09, *P* = 0.767). Particularly, fish fighting over territory with a nest exhibited overall shorter startle durations, and thus greater aggressive motivation, independently of acoustic conditions (*post-hoc*: *t*_1, 68_ = 1.38, *P* = 0.173; Fig. 3a), but those fighting over territory without a nest exhibited longer startle durations under noise than the control (*post-hoc*: *t*_1, 50_ = 2.52, *P* = 0.014; Fig. 3b). Furthermore, under the noise treatment animals were markedly less motivated to fight bigger opponents (exhibited longer startle durations; *post-hoc*: *t*_1, 59_ = 4.73, *P* < 0.001) than smaller opponents, but in control conditions they did not change their motivation with opponent size (*post-hoc*: *t*_1, 59_ = 1.64, *P* = 0.105; Fig. 3c).

**Fig. 4:**
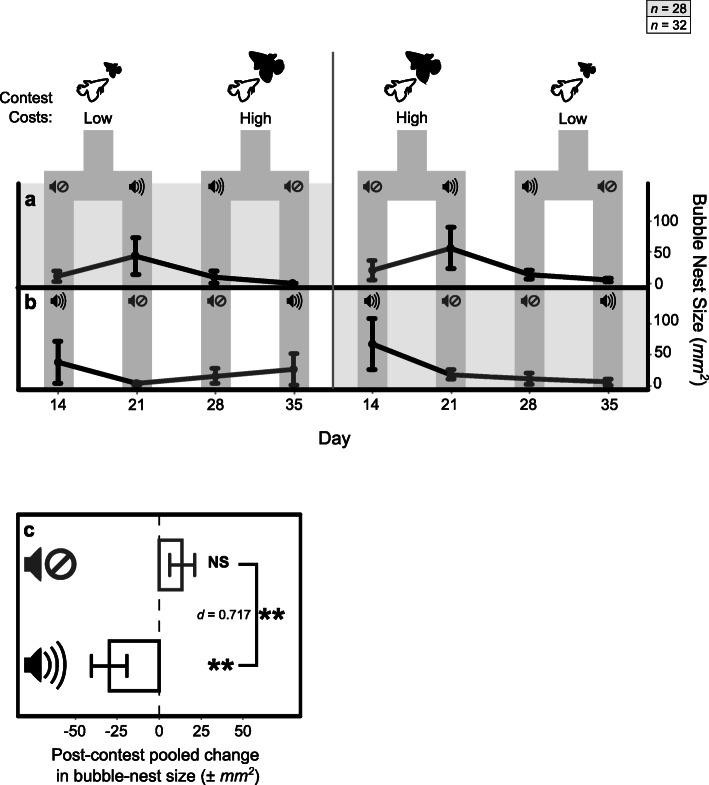
The effects of opponent size and acoustic condition on changes in bubble-nest construction. **a** Fish exposed first to control conditions exhibit increases in nest size and then decreases under repeated noise exposure until flooring, whereas **b** fish first exposed to noise exhibit decreases that persist under control conditions; in both scenarios effects are greater for fish that also faced bigger opponents first. **c** Comparisons of post-contest changes in nest size from pooled data across the 3 weeks show that following exposure to control conditions there is no significant change in bubble-nest size, but under noise conditions there is an overall decrease in size. Bars indicate the standard error and effect sizes are indicated by Cohen’s *d* where appropriate [**P ≤ 0.01]

Acoustic conditions during contests also affected territory use in terms of bubble-nest construction, where temporal changes in bubble-nest size (effect of days) varied significantly with differences in the order in which animals encountered noise conditions across contests (interaction: *χ*^*2*^_1, 119_ = 11.48, *P* = 0.009). Initial exposure to control conditions resulted in a steep increase in nest size followed by a steep decrease and flooring after subsequent repeated noise exposures (Fig. 4a). Conversely, initial exposure to noise resulted in a nest-size decrease and a maintenance of small-sized nests across subsequent repeated exposures to control conditions (Fig. 4b). This temporal effect of acoustic conditions was also modulated by opponent relative size during contests (3-way interaction: *χ*^*2*^_3, 119_ = 23.86, *P* < 0.001). In particular, the effects of temporal variation in acoustic conditions (Fig. 4a and b) were more pronounced when animals faced comparatively bigger opponents in their first two contests and comparatively smaller opponents in their last two contests (*R*^*2*^ = 0.298), in contrast to the opposite order of opponent sizes (*R*^*2*^ = 0.165).

Pooled changes in nest size in the week immediately after contests were not significantly affected by opponent size (*P* > 0.05), nor were these changes significantly different from no-change (*μ* = 0) when following contests with either smaller (*t*_44_ = − 0.68, *P =* 0.497) or bigger opponents (*t*_44_ = − 0.99, *P =* 0.326). However, these changes were significantly affected by acoustic conditions (*F*_1, 89_ = 7.06, *P =* 0.009; Fig. 4c), where a significant decrease in nest-size was noted after contests under noise (*t*_44_ = − 2.87, *P =* 0.006) but no significant change was noted after contests under control conditions (*t*_44_ = 1.84, *P =* 0.072).

## Discussion

Changes in the acoustic environment with the addition of artificial noise negatively influenced the motivation of *B. splendens* males to defend and subsequently use territory. However, in support of our hypotheses, effects depended on added costs from their opponent’s relative formidability and on reproductive benefits offered by nests in their territory. The first evidence comes from the effects of noise on fight motivation, which was decreased only in the absence of nests (Fig. 3a vs. b) and when opponents were relatively bigger than focal fish (Fig. 3c). This suggests that the effects of the acoustic environment are mediated by its influence on resource value, because information on resource value is expected to affect agonistic behaviour depending on the benefits or quality of the resource and the costs of a specific fight (e.g. the relative ability of the opponent [1]). Thus, here by experimentally manipulating the acoustic environment, we have demonstrated how information on an external stressor appropriates resource value and is integrated with opponent assessment to influence the motivation to defend territory (Fig. 1: *H*_*1*_).

On the one hand, bigger opponents are typically assessed as stronger and more likely to inflict injury [26, 50], in which case costs from fighting are greater and less worth incurring when the value attributed to a territory is low [7, 22], in this case without nests (Fig. 3a vs. b). On the other hand, the overall increased defence motivation of territory with nests, independently of the acoustic conditions (Fig. 3a), can be attributed to the hypothesised fitness value of bubble nests that exceeds resource and contest costs. This relies on the contribution of bubble nests to reproductive success by preparing fish for mating and constituting investment in parental care [27–30]. The effects of nest presence on contest motivation are in line with previous evidence showing that the defence of territory with nests is a paternal priority for male Siamese fighting fish [28, 31] and that bubble-nest holders exhibit elevated defensive aggressiveness against other males [32, 33]. In addition, the presence of physical elements in a territory is crucial for the strengthening of territorial associations in many species [51], which can offer an alternative explanation as to why motivation to defend territory is lower and more susceptible to noise in the absence of nests (Fig. 3b). Regardless of the underlying processes responsible for the effects of nest presence, the effect of the acoustic environment in the absence of nests suggests that the potential use of territories that have not yet been exploited is restricted by the perception and assessment of extrinsic stressors.

Additional evidence that the effects of noise are mediated by its influence on resource value comes from the effect of the acoustic environment during contests on subsequent motivation to construct bubble nests. Notably, individuals varied in their propensity to construct nests, as noted by the absence of nests in many occasions and the lack of individual variation in nest presence over time. However, for those that constructed nests, there was significant variation in their tendency to maintain and expand them across noise conditions. In line with our hypotheses, only after contests under the noise treatment was a decrease in nest-size observed, with the order in which fish were exposed to different acoustic conditions impacting nest-size changes over time (Fig. 4). Contrary to the increase in bubble nest size seen when fish have previously only experienced fights under control conditions, the persisting effects of an isolated exposure to the noise treatment (Fig. 4b), as compared to the decreasing effects of repeated exposures (Fig. 4a), might be explained by the greater degree of novelty at first encounters or one-off experiences. Unfamiliar aversive stimuli can result in more extensive stress responses, linked to neophobia and the management of uncertainty, when compared to the effects of aversive but familiar stimuli for which prior information has been collected and there is more certainty about related risks [52–54]. Although the reduction of effects on bubble-nest construction from repeated exposure to the noise treatment suggests that animals may habituate to noise (Fig. 4a), the slower decrease in construction is observed when average nest sizes are already at a minimum (day 21–28) and, thus, there is little room for further substantial decreases. In addition, within the period of our observations there is not enough evidence to conclusively demonstrate that effects reach a plateau or that they can be reversed following repeated exposure to the noise treatment. However, contrary to our laboratory setting, the option of alternative territories in the wild could mean that noisy territories are abandoned before animals are able to fully habituate to acoustic conditions [43]. Furthermore, behavioural and physiological responses to environments with novel and costly conditions can be more pronounced or prolonged in wild than captive-bred *B. splendens* [55].

Notably, the effects of temporal variation in noise on bubble-nest construction relied on their interaction with temporal variation in opponent size, which may relate to aggression effects on nesting [32, 33]. However, this effect is largely limited to initial exposure to sound conditions, where increases in nest size under control conditions without prior noise experience (Fig. 4a) and decreases in nest size after the first noise experience (Fig. 4a, post day 28, and 4b, post day 14) are greater when opponents are bigger. This suggests that external stress from noise, has a greater impact on the drive to use resources when those resources are contested by more formidable opponents, which supports our hypothesised noise effect on territory value based on cost-benefit interactions. When assessing pooled changes, opponent size had no significant immediate effects on nest construction, but nest-size change was affected by acoustic condition. In contrast to a weak increase in size under control conditions, a significant reduction in nest size was exhibited under noise treatment (Fig. 4c). Therefore, overall, noise elicits a decrease in bubble-nest construction or maintenance, but temporal nest-size changes also rely to a lesser extent on variations in relative opponent RHP across contests.

## Conclusions

Here we identify effects of the acoustic environment on territory value by demonstrating that the negative impact of noise on territory defence and use by *B. splendens* relies on interactions with added attainment costs (opponent RHP) and resource benefits (nest presence), as predicted by theory [7, 11, 26] (Fig. 1). Behavioural strategies in response to current conditions are context-specific (based on social and environmental factors) and informed by the evaluation of associated costs and benefits [56], but these strategies may become maladaptive under the unpredictability or novelty of some conditions, such as unprecedented noise from anthropogenic activity [57]. Following amassing evidence regarding the perceptual and cognitive capacities of non-human animals [58, 59], and indications that these capacities can be used when evaluating resources and assessing opponents [23], our findings highlight the need for new approaches to better understand the intricate ways in which human activity can influence fitness-related behaviour in other species. The impact on perceived or attributed value can have extensive effects on the desirability of resources and, thus, future studies are merited to further test this process both in the wild and under controlled manipulations.

## Methods

### Animals and housing

Adult male Siamese fighting fish *B. splendens* (*N* = 56) were acquired from a commercial supplier, Grosvenor Tropicals (Lisburn, Northern Ireland). Fish were bred and kept in captivity, but housed individually by the supplier as juveniles and adults, which addresses issues related to the degree of familiarity between them and the effects this might have on competition [60, 61]. After acquisition, animals were again housed individually in tanks (32 cm length × 22 cm width × 31 cm height) and visually isolated from their neighbours, to prevent stress-effects from aggressive interactions during housing and further limit familiarity effects. Housing tanks were filled with 15 L of water, enriched with plants, toys and shelter. Tank water was filtered, heated, aerated, and regularly tested for chemical quality, twice-weekly changed and kept at 26 ± 1 °C, 7.2 ± 0.4 pH and a regulated bacterial cycle. Further, regular health checks were carried out for signs of common diseases (as listed by Monvises et al. [62]). Each fish was kept on a moderate level diet of commercial feed (Hikari© Bio-gold; 4 pellets twice a day) with 38% animal protein, considered ideal in terms of growth and conversion rates [63]. On the day of experiments, animals were fed only after the staged contests to standardise motivation [1]. Light conditions were controlled at 12 h photoperiods (0700–1900), with experiments conducted during light periods (300 lx luminosity and 350–600 nm wavelength at water surface).

### Size measures

After a 72-h acclimation to laboratory conditions, measures of size were recorded for all fish to be used as estimates of RHP (see review by Arnott and Elwood [7]). First, the wet weight of each fish was calculated by the difference in mass between a water-filled container with and without the fish (cf Kareklas et al. [36]). Then fish were briefly removed from the container and placed with some water on a prepared waterproof surface marked by 1 cm squares. There, the anal and caudal fins of the fish were gently extended using a plastic pipette and fish were photographed using a suspended camera (*Sony HDR CX190E handy-cam* video camera) and immediately placed back into the water-filled container to be returned to their tank. Using Image-J, we used the 1 cm marked squares from the pictures as a reference for calibrating digital distance to actual distance (in cm) and used the software’s measurement tools for calculating standard body length (in cm: from tip of snout to the base of caudal peduncle) and the area of extended anal and caudal fins (cm^2^) for all fish.

Fin size and body length quantify morphological information that can be visually assessed in opponents during display [31], but each likely to provide separate information about an opponent’s RHP; length may designate overall size, but fin size may signal attack performance (e.g. charging and tail beating capacities [64]). Furthermore, preliminary analyses indicated that standard body-length and fin size (combined caudal and anal fin size) were only weakly related (*r*^*2*^ = 0.297; lack-of-fit: *r*^*2*^ = 0.572). Conversely, weight cannot be visually assessed but can provide a more inclusive measure of RHP because it can reflect the composite morphological state of animals, but may also reflect the private information fish have about their own physical capacities (i.e. their own perception of size or strength [22]). Indeed, weight strongly predicted standard length (*r*^*2*^ = 0.549; *F*_*56*_ = 65.76, *P* < 0.001) and combined fin size (*r*^*2*^ = 0.445; *F*_*56*_ = 43.31, *P* < 0.001) across all animals, focals and opponents. Although all animals were adults, weight may also reflect differences related to age and reproductive activity, but the relation here with length and fin size suggests that it at least represents morphological elements of RHP. Therefore, weight was used as an indicator of composite inter-contestant asymmetries in RHP and used for matching focal fish with comparatively bigger or smaller opponents, whereas length and fin size were considered as covarying factors that may predict added inter-individual size-related effects and visual-assessment biases. The mean relative weight difference of opponents compared to focals was 0.38 g ± 0.56 SD and ranged between − 0.64 g and + 1.63 g.

### Experimental procedures

Focal fish (*n* = 30) were housed in their individual tanks for 2 weeks before experiments to ensure territorial establishment via the use of landmarks in their tank [51] and to allow time for bubble-nest building. The experiment consisted of four weekly contests so that focal fish faced both a bigger and a smaller opponent, each under both noise and control acoustic conditions (a within-individual 2 × 2 factorial design), with acoustic-treatment order being randomised and opponent-size order counterbalanced across individuals. Opponent size was manipulated by replacing opponents between contests, based on their relative weight. Acoustic conditions were manipulated using a soundproofed experimental lid with an embedded speaker (Fig. 2a), which either played white noise under treatment or remained silent during controls (see similar protocols of air suspended speakers eliciting response in related anabantoid fish [47, 65]). Focal and opponent tanks were kept with some distance between them (~ 2 cm) to control for sound vibration effects on opponents. White noise treatments resembled distant-traffic noise, low-pass filtered to 100 Hz frequency with 6 dB kHz^− 1^ decrease towards higher levels [66] (set to 80 dbA at 1 m, SL-100 Voltcraft, Hirschau meter). Underwater sound recordings were performed using a hydrophone (Aquarian Audio H2a-XLR; omnidirectional; sensitivity: -180 dB re. 1 V/μPa), placed centred on the bottom of a tank (conservative measure of greatest depth from source) and analysed in the Audacity® software. Compared to controls, the noise treatment exhibited markedly greater changes in sound pressure level (Fig. 2b) and five-fold increases in frequency (Fig. 2c), kept within the 5 kHz maximum hearing threshold attributed to *B. splendens* based on its auditory structure similarities to other anabantoid fish, such as *Trichopsis spp* [47, 48]. The maximum of ~ 40 dB change in sound pressure levels at frequencies of 1–4 kHz (Fig. 2d) under noise treatment reflected nominal low-magnitude underwater noise in areas near terrestrial city traffic or with light shipping [49, 67] and matched profiles eliciting response in other gouramis (maxima of 5 kHz; 20–40 dB changes [47, 68, 69]). The lid and noise conditions were experienced by focal animals for 10 min before contests, allowing for both acclimation to the lid and assessment of noise conditions, and maintained only for the duration of each staged contest. The lid of housing tanks was opaque and maintained on opponent tanks during contests to standardise lighting conditions after placing the soundproofed experimental lid.

Before each contest, the water surface of tanks was photographed for recording bubble nests on the water surface. Contests were staged in the form of simulated territorial intrusions by removing opaque covers that visually isolated stimulus fish (*n* = 26) in immediately neighbouring tanks (acclimated overnight). The onset of contests was set at the first agonistic behaviour by focal fish, either in terms of bite attacks, tail beats, or display (either *frontal display* - presenting extended gills to opponent- or *lateral display* - presenting side with flared fins to opponent [31]). Fight motivation was probed by measuring focal recovery times from startles elicited by dropping a glass marble (24 g) through one of the two bilateral tubes in the lid, with the side counterbalanced across individuals and consecutive contests, and between within-contest consecutive probes. The marble landed in the water surface from a 10 cm height and invoked a startle response in focal animals via visual and mechanosensory cues from the distinct splash (Fig. 2a [36–39];). This probe was performed twice during contests, at 5 min after onset of contests and again at 5 min after focal fish resumed interaction. Following recovery from the second startle, fish were allowed to interact for a final 5 min period before concluding the test by replacing the opaque cover between tanks. Although the overall duration of the staged events varied due to differences in startle durations, the initial 5 min interaction and the two 5 min interactions after each probe ensured that all contests had a standard 15 min interaction between focals and opponents. Contests were video recorded (*Sony HDR CX190E handy-cam* video camera) and the experimenter remained hidden during test-related manipulations by a large cover (150 cm × 150 cm) and away in interim periods, to minimise interference.

### Behavioural measures

From the pictures taken before each contest, we first recorded the presence of bubble nests and then measured nest size (in mm^2^) using the Image-J software to calibrate digital to actual area. Nest-presence was identified in overall 69 contests: 37 out of 60 under control conditions (19 against bigger opponents, 18 against smaller opponents) and 32 out of 60 under noise treatment (18 against bigger opponent, 14 against smaller opponents). This suggests that fish had at least some underlying differences in reproductive state, but our within-individual experimental design controlled for the influence of this on variations between contests. Further, there were no temporal effects (Binary; ID × Day: *χ*^*2*^_*3, 119*_ = 0.31, *P* = 0.957) or noise effects (ID × Noise: *χ*^*2*^_*3, 119*_ = 0.10, *P* = 0.758) on individual nest-presence, indicating that the tendency of individuals to construct nests or not was consistent across the experimental period and independently of acoustic condition. This tendency could be attributed to other factors, which according to evidence could include stressors such as temperature or water quality [70, 71], but these parameters were controlled and standardised in our study. In addition, alternative effects from individual differences in baseline stress levels are unlikely to have a direct impact, but could influence the relationship between body size and nest quality [70, 71]. However, mean weight did not differ between males that tended to build nests and those that did not (*t* = 0.66, *P* = 0.513), and there was no significant relationship between nest size and body size (length: *r* = 0.088, *P* = 0.340; weight: *r* = 0.081, *P* = 0.381). On the contrary, the size of nests exhibited individual differences over time (ID × Day: *χ*^*2*^_*3, 119*_ = 444.38, *P* < 0.001). We therefore, used the size of nests as an indicator of individual changes in construction across contests, with bubble nest size before each contest used to calculate differences in construction between weeks (± mm^2^), for examining changes in territory use over time. Because the absence of nests was set at 0 mm^2^, the variation reflected changes driven by fish who tended to construct nests.

From the recordings of contests, the time taken to recommence display following each of the two startle probes was used as an estimate of aggressive motivation, with shorter recovery times (in seconds) indicating greater aggressive motivation [36–39]. The combined duration of frontal and lateral display across the contest was also measured, both for focals and opponents. Overall, fish exhibited display across all contests and total display duration was significantly related to weight (*r* = 0.310, *P* < 0.01), indicating that weight was a robust predictor of RHP. In addition, opponent display duration was not affected by the acoustic conditions in the focal tank (*t*_*1,119*_ = − 0.22, *P* = 0.826), which suggests that noise was largely contained to the focal tank and did not affect the behaviour of opponents during contests. The startle durations of focals were strongly predicted by display durations (*R*^*2*^ = 0.396, *F*_*1, 119*_ = 77.43, *P* < 0.001), confirming their representation of aggressive motivation. Although startle durations increased (*t*_*1,119*_ = 5.90, *P* < 0.001) between the first (7.52 s ± 7.98 SD) and second probe (26.49 s ± 39.88 SD), a likely effect of energetic expenditure [7, 72], variation in startle duration was consistent between probes (Pearson, *r* = 0.650, *P* < 0.001) indicating consistency in inter-individual differences. Therefore, the mean startle duration of the two probes from each contest was used as a measure of individual aggressive motivation in statistical tests.

### Statistical analysis

Data calculations, statistical tests and graphical representations were carried out in the statistical software Minitab® version 17 (Minitab Inc., State College, PA, USA) and SPSS version 22 (IBM Corp., Armonk, NY. USA). Normality was tested for both raw data and residuals from the models (Shapiro-Wilk) to inform the choice and fitness of statistical tests. Across tests, Cohen’s *d* was used as a measure of effect size, based on the mean and standard deviation, and *R*^*2*^ as an effect size measure for interactions with temporal changes.

According to our first two hypotheses, a decrease in territory value under noise is expected to reduce defence motivation (1) when facing bigger opponents that pose higher costs but (2) not when a nest is present, because it increases territory benefits. Therefore, we first tested whether mean startle durations per contest, our measure of defence motivation, was affected by the interaction of acoustic condition (noise playback or control) with relative opponent RHP (bigger or smaller in weight than focal) and nest presence. For this a mixed effects model with a log-link function was used because startle duration data was not normally distributed (*P* < 0.01), but residuals from the model conformed to normality (*P* > 0.05). The model included opponent length and fin size as covariates to control for assessment biases from visual signals [36, 39], as well as day and individual identity of focals and opponents as random factors to respectively control for carry-over effects and pseudoreplication. *Post-hoc* pairwise comparisons (Fisher’s LSD) were then performed to examine differences between contests under different conditions.

According to our third hypothesis, (3) a decrease in territory value under noise is expected to also reduce subsequent motivation to use the territory for nest-building, which may include interaction effects with opponent RHP, where fighting relatively bigger opponents imposes added costs. As such, we tested the effects of acoustic conditions and relative opponent size on both temporal variation in bubble-nest size across contests, to identify carry-over effects from sequential changes [73], and immediate nest-size changes following each contest. For effects on the temporal nest-size variation, a Gamma model with a log-link function was used on the bubble-nest size data to address their skewness towards zero due to the high no-nest incidence (${\overset{\sim }{\mu}}_3$ > 3.69) and their consequent lack of normality (*P* < 0.005). Recent evidence [74] shows that skewness violations do not significantly contribute to estimate violation in mixed-type models, especially when data are not severely skewed $\Big({\overset{\sim }{\mu}}_3$ < 3). The residuals from the model were in line with this by exhibiting notable decreases in skewness (${\overset{\sim }{\mu}}_3$ = 1.77) and increases in normality (*P* = 0.01). This model was used to test for variation over time (days in housing tank) and whether this was affected by acoustic treatment order and its interaction with opponent-size order across contests, where focal length was included as a covariate (to control for body-size effects on bubble-nest construction [28, 70, 71];) and individual identity was included as a random factor (to control for pseudoreplication). For the immediate effects, we pooled calculated changes in nest size for the week following each contest (normally distributed) and used an ANOVA model to test whether these changes relied on acoustic condition (noise playback or control) and relative opponent RHP (bigger or smaller in weight than focal) during the contest, including post hoc one-sample *t*-tests for assessing significant differences from no change under different conditions.

## Supplementary Information

**Additional file 1: Table S1**. All data used in experimental and preliminary analyses.

## Data Availability

All data generated or analysed during this study are included in this published article and its supplementary information files.
